# PRK1/PKN1 controls migration and metastasis of androgen-independent prostate cancer cells

**DOI:** 10.18632/oncotarget.2653

**Published:** 2014-12-10

**Authors:** Cordula A. Jilg, Anett Ketscher, Eric Metzger, Barbara Hummel, Dominica Willmann, Vanessa Rüsseler, Vanessa Drendel, Axel Imhof, Manfred Jung, Henriette Franz, Stefanie Hölz, Malte Krönig, Judith M. Müller, Roland Schüle

**Affiliations:** ^1^ Urologische Klinik und Zentrale Klinische Forschung, Klinikum der Universität Freiburg, Freiburg 79106, Germany; ^2^ University of Freiburg, Faculty of Biology, Freiburg 79104, Germany; ^3^ Universitätsklinikum Köln, Institut für Pathologie, Köln 50937, Germany; ^4^ Department of Pathology, University Medical Center, Freiburg, Germany; ^5^ Adolf-Butenandt Institute and Munich Center of Integrated Protein Science (CIPS), Ludwig-Maximilians-University of Munich, Munich 80336, Germany; ^6^ Institute of Pharmaceutical Sciences, University of Freiburg, Freiburg 79104, Germany; ^7^ BIOSS Centre of Biological Signalling Studies, Albert-Ludwigs-University, Freiburg, Germany; ^8^ Deutsches Konsortium für Translationale Krebsforschung (DKTK), Standort Freiburg, Germany

## Abstract

The major threat in prostate cancer is the occurrence of metastases in androgen-independent tumor stage, for which no causative cure is available. Here we show that metastatic behavior of androgen-independent prostate tumor cells requires the protein-kinase-C-related kinase (PRK1/PKN1) *in vitro* and *in vivo*. PRK1 regulates cell migration and gene expression through its kinase activity, but does not affect cell proliferation. Transcriptome and interactome analyses uncover that PRK1 regulates expression of migration-relevant genes by interacting with the scaffold protein sperm-associated antigen 9 (SPAG9/JIP4). SPAG9 and PRK1 colocalize in human cancer tissue and are required for p38-phosphorylation and cell migration. Accordingly, depletion of either ETS domain-containing protein Elk-1 (ELK1), an effector of p38-signalling or p38 depletion hinders cell migration and changes expression of migration-relevant genes as observed upon PRK1-depletion. Importantly, a PRK1 inhibitor prevents metastases in mice, showing that the PRK1-pathway is a promising target to hamper prostate cancer metastases *in vivo*.

**Statement of significance:**

Here we describe a novel mechanism controlling the metastatic behavior of PCa cells and identify PRK1 as a promising therapeutic target to treat androgen-independent metastatic prostate cancer.

## INTRODUCTION

Prostate cancer (PCa) is the second leading cause of cancer deaths in men worldwide [1]. Localized PCa can be cured by surgery and/or radiotherapy. However, depending on initial tumor stage, biochemical and clinical relapse occurs in 20–53% of the cases within the first five years [2]. Due to the hormone-dependent properties of PCa, the majority of men with relapsing PCa initially responds to hormonal depletion but will inevitably develop androgen-independent PCa over time, followed by first regional and later distant metastases. For this stage of disease there is no curative therapy available. Therefore, the most common cause of clinical complications and subsequent death originates from metastases. Thus, this clinical dilemma urgently calls for new therapeutic strategies to treat advanced PCa.

Hormone-dependent proliferation of PCa cells, can be blocked by depletion of the protein-kinase C-related kinase 1 (PRK1/PKN1) as exemplified in LNCaP cells [3]. In the scenario of metastatic androgen-independent PCa, the role of PRK1 is only poorly understood. The serine/threonine kinase PRK1 can be activated by the Rho family of small G proteins, thereby mediating Rho-dependent signaling pathways [4]. PRK1 is able to control various processes such as regulation of the intermediate filaments, the actin cytoskeleton, cell migration, and tumor cell invasion [5, 6].

Formation of metastases requires active tumor cell migration and invasion [7]. Current knowledge implies that in cancer cells integrins together with receptor thyrosine kinases confers positional control of migrating tumor cells for the attachment to the extracellular matrix (ECM) [8]. Integrin adaptors such as enhancer of filamentation/neural precursor cell expressed developmentally down-regulated 9 (NEDD9) influence signaling pathways, actin cytoskeleton reorganization, and ECM degradation [8, 9].

Furthermore, regulation of cell motility, site-specific extracellular signaling and cell protrusion, is controlled by focal adhesion (FA) [7]. FA are built of several proteins including paxillin (PXN), talin, and zyxin. Overexpression of proteins found in FA increases migration and/or metastasis in various types of cancer [10, 11].

In addition to tumor cell motility and extracellular attachment of tumor cells, unoffended survival of tumor cells by masking them from the natural immune system is crucial for tumor progression. Cancer cells foster a tolerant microenvironment and activate immunosuppressive mechanisms [12]. Ectonucleoside triphosphate diphosphohydrolase 1 (ENTPD1/CD39) and 5′-nucleotidase (NT5E/CD73) are located in the tumor cell membrane and impair antitumor T-cell responses by converting adenosinetriphosphate to adenosine. Increasing extracellular adenosine level comprises a pivotal axis in tumor immune escape [13, 14].

Scaffold proteins, operating on substrate specificity and cellular localization of proteins influence the signaling cascades such as the mitogen activated protein kinase (MAPK) pathway which in known to be important in cancer pathways [15]. JNK-interacting proteins (JIP1, JIP2, JIP3) act on c-Jun N-terminal kinase (JNK)-signaling, whereas SPAG9/JIP4 modulates p38 (MAPK14) signaling [15–17]. In the nucleus, the serine/threonine kinase p38 phosphorylates and thereby activates transcription factors, such as the ETS domain-containing protein Elk-1 (ELK1) [18, 19]. In human breast cancer cells, ELK1 is crucial for the initiation and progression of metastatic processes [20, 21].

Here, we show that PRK1 controls migration and invasion of androgen-independent PCa cells *in vivo* and *in vitro*. Transcriptome analysis revealed PRK1-regulated genes such as *NT5E*, *NEDD9*, and *PXN*, illustrating the migratory signature of PRK1. We identified SPAG9 as novel binding partner required for proper signaling of PRK1 to p38. ELK1, a p38 effector, controls the transcription of migration-relevant genes such as *NT5E*, *NEDD9*, and *PXN* that characterize the phenotype of PRK1 depletion. *In vivo* metastasis of androgen-independent prostate cells is hampered by a clinically approved PRK1 inhibitor. Taken together, we describe a novel mechanism controlling the metastatic behavior of PCa cells and identify PRK1 as a promising target to prevent metastasis of androgen-independent PCa.

## RESULTS

### PRK1 controls migration and invasion of androgen-independent PCa cells

**Figure 1:**
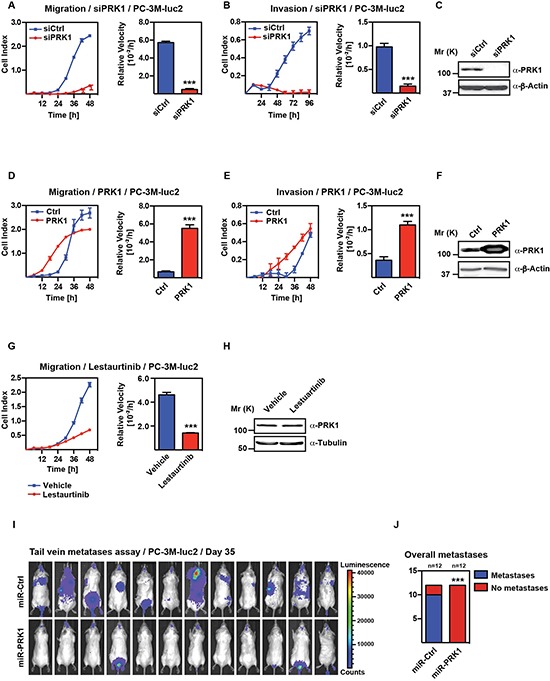
PRK1 controls migration and invasion of androgen-independent prostate cancer cell lines and determines development of metastases *in vivo* Migration **(A)** and invasion **(B)** assays of PC-3M-luc2 cells treated with siRNA against PRK1 (siPRK1) or unrelated siRNA (siCtrl). Migration **(D)** and invasion **(E)** assay of PC-3M-luc2 stable overexpressing PRK1 or control transfected cells. (G) Migration of PC-3M-luc2 cells treated with PRK1-inhibitor Lestaurtinib (25 μM). (A, B, D, E, G) Cell indices and relative velocities are shown. **(C, F, H)** Levels of PRK1 were analyzed by Western blots decorated with the indicated antibodies. β-Actin was used as a loading control. *n* ≥ 3. **(I)** PRK1 knockdown abolished development of overall metastases in tail vein metastases assay. Bioluminescent signal of immunodeficient mice at day 35 after injection of PC-3M-luc2 cells either stably expressing miRNA against PRK1 (miR-PRK1) or control (miR-Ctrl). Statistics for comparing overall metastases **(J)** was done using Fisher′s exact test. Error bars represent ± SD or + SD. *** *p* ≤ 0.001.

To investigate the role of PRK1 during metastatic processes of PCa we first analyzed whether modulation of PRK1 levels in androgen-independent prostate tumor cell lines such as PC-3M-luc2 and Du145 influences migration and invasion. In both cell lines migration and invasion was strongly decreased upon knockdown of PRK1 compared to cells treated with unrelated control siRNA (Figure 1A-C, Supplemental Figure 1C-E). Decreased migration of PC-3M-luc2 cells was also observed upon stable transfection of miRNA directed against a different region of PRK1 (Supplemental Figure 1A and 1B) supporting the significance of the effect after PRK1 depletion.

In comparison, PRK1 knockdown lead to a minor but not significant impairment of proliferation of PC-3M-luc2 and Du145 cells (Supplemental Figure 1F-H).

Furthermore, caspase-3-apoptosis assay in PC-3M-luc2 cells showed an unchanged rate of apoptosis (Supplemental Figure 1I). We also observed decreased migration upon knockdown of PRK1 in the MDA-MB-231 triple negative breast cancer cell line (Supplemental Figure 1J and 1K) whereas no effect on migration was seen upon PRK1 depletion in a pancreas carcinoma cell line PANC-1 (Supplemental Figure 1L and 1M). Conversely, stable overexpression of PRK1 increased migration and invasion of PC-3M-luc2 cells (Figure 1D-F). To analyze whether the decreased migration after PRK1 knockdown depends on the enzymatic function of PRK1, we treated PC-3M-luc2 cells with the PRK1- inhibitors Lestaurtinib or Ro318220. Upon treatment with both inhibitors a significant reduced migration of PC-3M-luc2 was observed (Figure 1G and 1H, Supplemental Figure 1N and 1O). We also confirmed the impairment of migration of Du14 cells after treatment with Ro318220 (Supplemental Figure 1P and 1Q). Although Lestaurtinib is an inhibitor for FLT3, JAK2, TrKA, TrKB, TrKC, and PRK1 [22] neither FLT3 nor TrKA-C are expressed in PC-3M-luc2 cells (Supplemental Figure 1R) nor treatment with the JAK-inhibitor CP-690550 showed an effect on migration (Supplemental Figure 1S). We therefore concluded that the inhibition of migration by Lestaurtinib in PC-3M-luc2 cells is indeed due to PRK1 inhibition. Taken together, these data show that PRK1 controls migration and invasion, but not proliferation in androgen-independent PCa cells through its kinase function.

### PRK1 controls metastasis *in vivo*

To further support the role of PRK1 in metastatic processes we used an *in vivo* metastasis mouse model, in which PCa cells extravasate from the venous blood system to form distant metastases. PC-3M-luc2 cells, stably expressing miRNA targeting PRK1 were injected into the lateral tail vein of immunodeficient mice, imitating metastatic tumor cell spreading (Supplemental Figure 1T). Metastases formation was monitored by bioluminescence over 5 weeks and verified by histological staining (Supplemental Figure 1U). Development of metastases was robustly abolished upon PRK1 depletion compared to control, demonstrating the biological relevance of PRK1 to promote metastasis *in vivo* (Figure 1I and 1J).

### PRK1 determines migration by regulation of *NT5E, NEDD9*, and *PXN*

**Figure 2:**
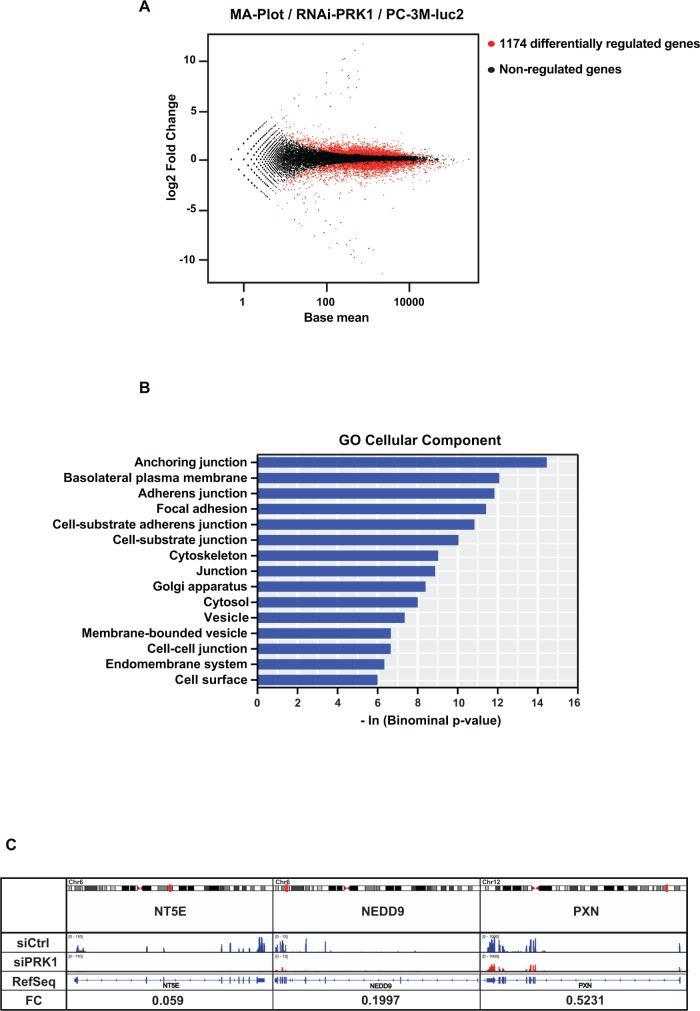

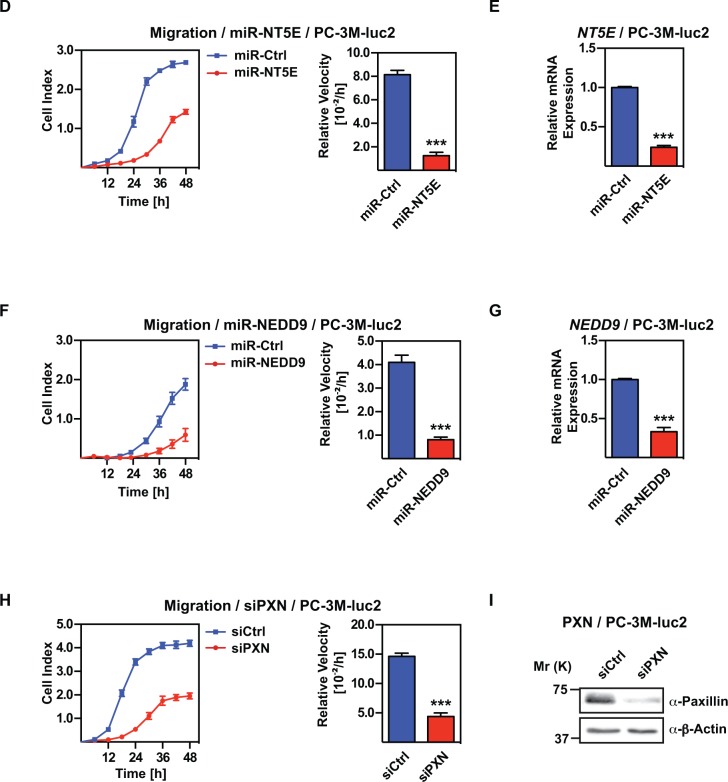
PRK1 regulates transcription of genes determining migration and invasion **(A)** MA-Plot representing the differentially regulated genes (red dots) in PC-3M-luc2 cells upon knock down of PRK1. **(B)** DAVID analysis for GO “cellular component” for differentially regulated genes with a *p*-value < 10^−3^. **(C)** Read coverage displaying the downregulation of *NT5E*, *NEDD9*, and *PXN* upon PRK1 knockdown. FC: Fold Change. **(D, F)** Migration assays of PC-3M-luc2 cells stable transfected with miRNA against *NT5E* (miR-NT5E), *NEDD9* (miR-NEDD9), or unrelated control miRNA (miR-Ctrl). **(E, G)** Efficiency control of knockdown. mRNA levels of *NT5E* or *NEDD9* were analyzed by qRT-PCR. **(H)** Migration assay of PC-3M-luc2 cells treated with siRNA against PXN (siPXN) or unrelated siRNA (siCtrl). **(I)** Efficiency control of knockdown. Protein expression of PXN was analyzed by Western blots. (D, F, H) Cell indices and relative velocities are shown. *n* ≥ 3. Error bars represent ± SD or + SD. *** *p* ≤ 0.001.

To identify genes that mediate the effect of *PRK1* on migration and invasion we performed transcriptome analyses. RNA isolated from PC-3M-luc2 cells that were either treated with siRNA against PRK1 or unrelated control siRNA was subjected to massive parallel RNA-sequencing (RNA-seq). Bioinformatic analyses revealed that in total 1174 genes were differentially regulated upon knockdown of PRK1 (Figure 2A). For further characterization of the 1174 genes we subjected them to DAVID gene ontology analysis. In accordance with the migratory phenotype the “cellular component” analysis revealed the presence of genes associated with anchoring junctions, basolateral plasma membrane, adherence junctions, and focal adhesions (Figure 2B).

Of note genes such as PXN and NEDD9 are abundantly comprised in the regulated genes of the term “anchoring junction” and “focal adhesions” in the DAVID analysis. NT5E was strongly repressed by PRK1 knockdown in the RNA-seq (0.059-fold; *p*-value: 4.1 × 10^−93^) (Figure 2C). Consequently, we hypothesized that these 3 genes could be prototypical representatives mediating the PRK1 migratory phenotype. Indeed, verification by qRT-PCR revealed that expression of *NT5E*, *NEDD9*, and *PXN* was severely downregulated by either PRK1 RNAi in PC-3M-luc2 or DU145 cells or the PRK1 inhibitors Ro318220 or Lestaurtinib in PC-3M-luc2 (Supplemental Figure 2A-D). Conversely, expression of *NT5E*, *NEDD9*, and *PXN* were significantly upregulated upon PRK1 overexpression in PC-3M-luc2 cells (Supplemental Figure 2E). To provide further evidence that these genes are mediators of PRK1-controlled migration, we depleted PC-3M-luc2 of *NT5E*, *NEDD9*, or *PXN* either by siRNAs against PXN or stable expression of miRNAs against NEDD9 and NT5E. Knockdown of either *NT5E*, *NEDD9*, or *PXN* severely impaired migration of PC-3M-luc2 cells (Figure 2D-I) supporting the significance of the effect after *NT5E*, *NEDD9*, or *PXN* depletion. In summary, our transcriptome analysis, as exemplified by the representative genes *NT5E*, *NEDD9*, and *PXN*, revealed that PRK1 is a key regulator of genes responsible for migration in PC-3M-luc2 cells.

### PRK1 associates with SPAG9 in PC-3M-luc2 cells

**Figure 3:**
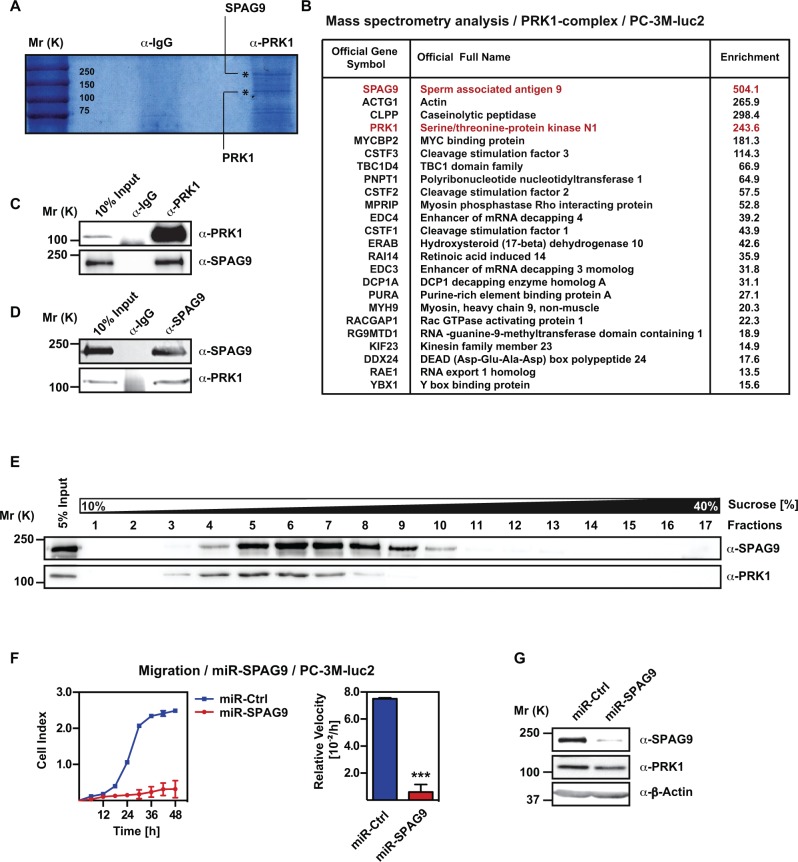

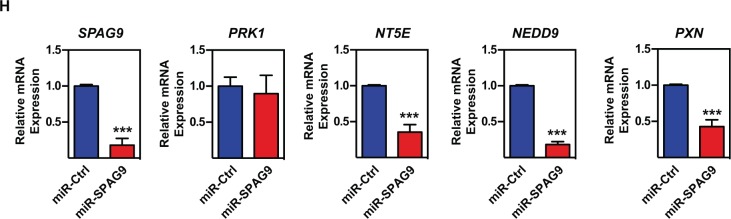
PRK1 associates with SPAG9 in PC-3M-luc2 cells **(A)** Coomassie-staining of SDS-page after immunoprecipitation with anti-PRK1-antibody. **(B)** PRK1 interacting proteins identified by mass spectrometry. **(C, D)** Western blots showing coimmunoprecipitation of endogenous PRK1 with SPAG9. **(E)** PRK1 in complex with SPAG9 verified by sucrose gradient centrifugation. Western blot were decorated with the indicated antibodies. **(F)** Migration assays of PC-3M-luc2 cells stable expressing miRNA against SPAG9 (miR-SPAG9) or control (miR-Ctrl). **(G)** Levels of SPAG9 and PRK1 were analyzed by Western blot. **(H)** Efficiency control of *SPAG* knockdown and expression of “signature genes”. mRNA levels of *SPAG9*, *PRK1*, *NT5E*, *NEDD9*, and *PXN* were analyzed by qRT-PCR. **(F)** Cell index and relative velocities are shown. *n* ≥ 3. Error bars represent ± SD or + SD. *** *p* ≤ 0.001.

To get insights into the mechanism of PRK1 signaling and gene regulation, we analyzed which endogenous proteins assemble with PRK1 in PC-3M-luc2 cells (Figure 3A). Thus, we performed immunoprecipitation with an anti-PRK1-antibody using PC-3M-luc2 cell extracts followed by mass spectrometry analysis. Among the identified proteins SPAG9/JIP4 (sperm associated antigen 9) was the most enriched protein (Figure 3B). We confirmed the interaction of endogenous PRK1 and SPAG9 by coimmunoprecipitation (Figure 3C and 3D) and by cofractionation in a sucrose gradient centrifugation (Figure 3E). Immunofluorescence staining revealed the cytoplasmatic colocalization of PRK1 and SPAG9 in PC-3M-luc2 cells (Supplemental Figure 3A-H). Taken together we identified SPAG9 as a new interacting protein of PRK1.

### PRK1 regulates migration through assembling with SPAG9

To investigate whether PRK1-controlled migration requires SPAG9 we analyzed stably transfected PC- 3M-luc2 expressing two different miRNAs against SPAG9. Knockdown of SPAG9 leads to dramatically reduced migration compared to control (Figure 3F and 3G, Supplemental Figure 3I and 3J). Next, we asked whether SPAG9 affects expression of the PRK1-dependent migration-relevant genes. Expression of *NT5E*, *NEDD9*, and *PXN* was downregulated in PC-3M-luc2 cells depleted of SPAG9, similar to PRK1 knockdown (Figure 3H). In summary, these data show the importance of SPAG9 for the migration and regulation of PRK1 target genes.

### PRK1 and SPAG9 influence phosphorylation of MAPK14 (p38)

**Figure 4:**
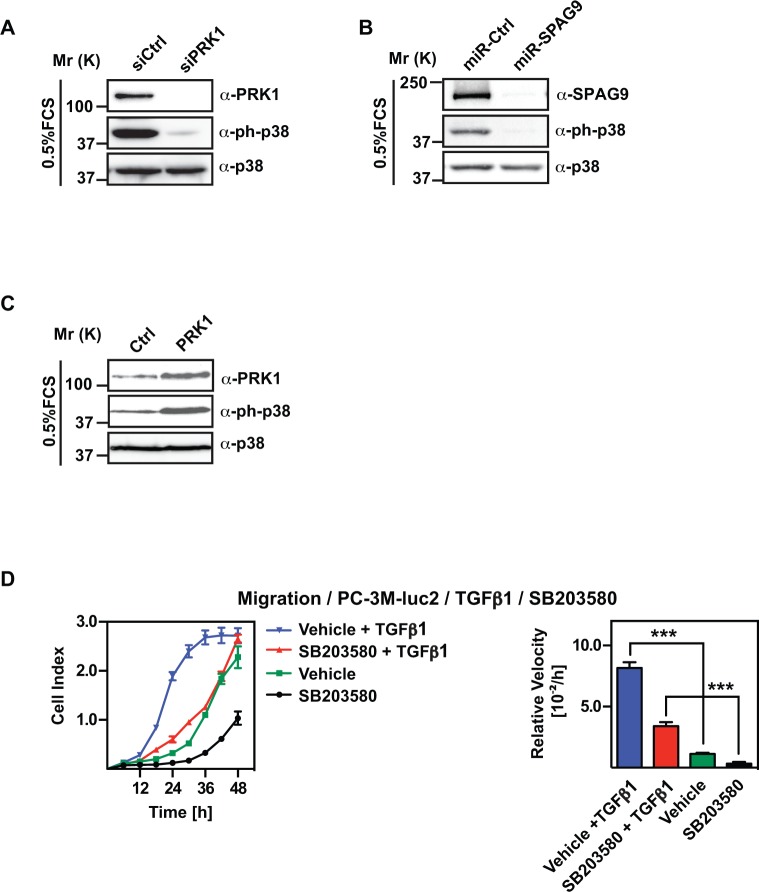

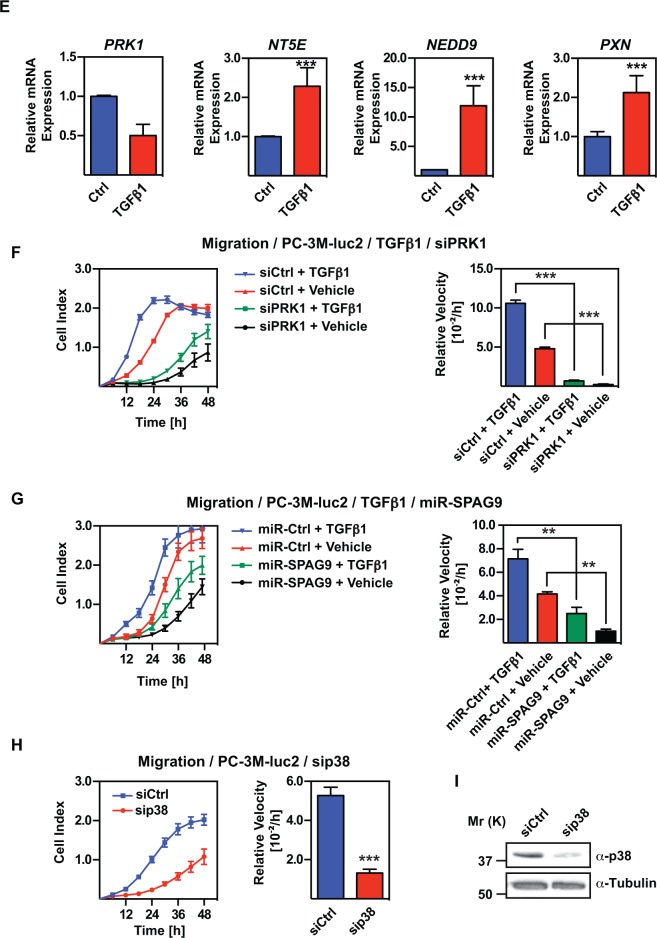

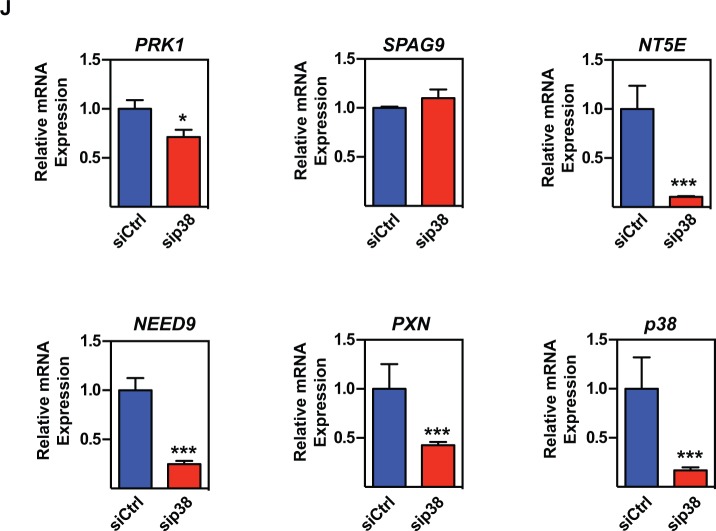
PRK1 and SPAG9 determine phosphorylation status of p38 **(A, B)** Western blots showing levels of phospho-p38 (ph-p38) upon knockdown of PRK1 or SPAG9. **(C)** Level of phospho-p38 was analyzed in Western blots with the indicated antibodies upon overexpression of PRK1 in PC-3M-luc2 cells. Total amount of p38 protein is shown as control. **(D)** Migration assay of PC-3M-luc2 cells treated with either TGFβ1 (2ng/ml), or the p38 inhibitor SB203580 (20 μM) or both compared to control vehicle. **(E)** mRNA levels of *PRK1*, *NT5E*, *NEDD9*, and *PXN* upon treatment of PC-3M-luc2 with TGFβ1 (2 ng/ml) were analyzed by qRT-PCR. **(F)** Migration assay of PC-3M-luc2 cells treated with TGFβ1 or vehicle after knockdown of PRK1 (siPRK1) or control (siCtrl). **(G)** Migration assay of PC-3M-luc2 cells treated with TGFβ1 or vehicle after miRNA-mediated knockdown of SPAG9 (miR-SPAG9) versus control (miR-Ctrl). **(H)** Migration assay of PC-3M-luc2 treated either with siRNA against p38 (sip38) or unrelated control siRNA (siCtrl). **(I)** Efficiency control of p38 knockdown was performed by Western blot analysis. **(J)** mRNA levels of *PRK1*, *SPAG9*, *NT5E*, *NEDD9*, *PXN*, and *p38* upon knockdown of p38 in PC-3M-luc2 cells analyzed by qRT-PCR. (D, F, G, H) Cell indices and relative velocities are shown. *n* ≥ 3. Error bars represent ± SD or + SD. ** *p* ≤ 0.01, *** *p* ≤ 0.001.

Both, PRK1 and SPAG9 have been described in the regulation of MAPK14 (p38) signaling [16, 23, 25]. Phosphorylation of p38 is necessary for activation and further downstream signaling events such as phosphorylation of transcription factors [15]. To validate that PRK1 and SPAG9 cooperate to facilitate p38-activation in PC-3M-luc2 cells we studied the phosphorylation level of p38 at Thr180/Tyr182, indicating active p38. Western blot analysis of PC-3M-luc2 cell lysate revealed that upon depletion of PRK1 or SPAG9, phosphorylation of p38 at Thr180/Tyr182 (phospho-p38) decreased significantly (Figure 4A and 4B). Moreover, we confirmed in Du145 cells that p38ph-level is reduced after PRK1 knockdown (Supplemental Figure 4A). Conversely, overexpression of PRK1 in PC-3M-luc2 increased the level of phospho-p38 (Figure 4C).

Next, we asked for the relevance of phospho-p38 for the migratory behaviour of PC-3M-luc2 cells. Previous studies showed that TGFβ1 treatment led to strong phosphorylation of p38, whereas enzymatic inhibition of p38 by SB203580 blocked p38-signaling [19, 26]. TGFβ1 treatment caused a significant increase, whereas treatment with SB203580 lead to decreased migration of PC- 3M-luc2 cells (Figure 4D). Upon TGFβ1 treatment were observed a significant upregulation of *NT5E*, *NEDD9*, and *PXN* expression, the genes which we previously identified as “signature genes” for PRK1-dependent migration (Figure 4E).

Next, we asked whether PRK1 or SPAG9 depletion effects TGFβ1-promoted migration. PC-3M-luc2 cells were transfected with siRNA against either PRK1 or SPAG9 and subsequently treated with TGFβ1. Importantly, the pro-migratory effect of TGFβ1 was no longer observed upon either knockdown of PRK1 (Figure 4F) or SPAG9 (Figure 4G), corroborating the requirement of PRK1 and SPAG9 for TGFβ1 signaling to p38. Finally, depletion of p38 not only impairs migration in PC-3M-luc2 (Figure 4H and 4I) but also expression of *NT5E*, *NEDD9*, and *PXN* (Figure 4J). We confirmed in Du145 cells that knockdown of p38 impairs cell migration and mRNA expression of *NT5E*, *NEDD9*, and *PXN* (Supplemental Figures 4B-D). Taken together, these data demonstrate that PRK1 and SPAG9 are necessary for the p38-mediated migratory capability of PC-3M-luc2 cells.

### ELK1 targets PRK1 migratory signature genes

**Figure 5:**
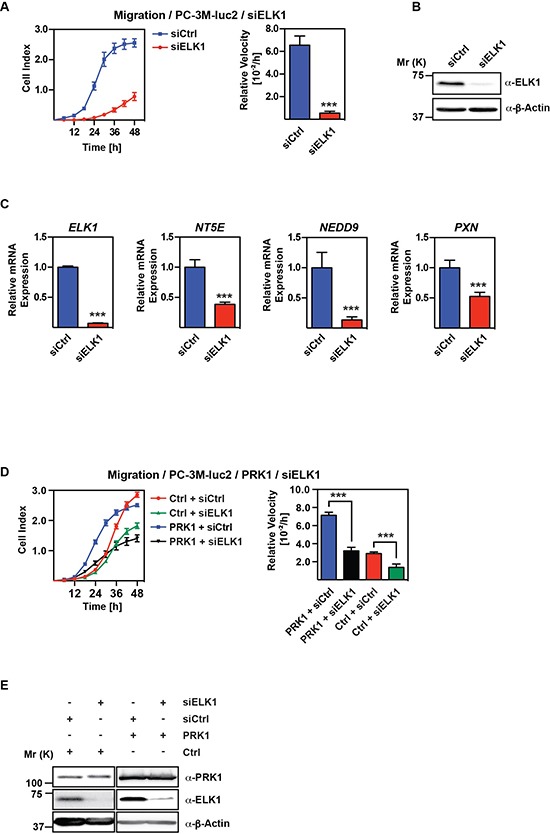
ELK1 regulates migration and occupies PRK1-regulated genes **(A)** Migration assay of PC-3M-luc2 cells treated either with siRNA against ELK1 (siELK1) or unrelated control siRNA (siCtrl). **(B)** Verification of ELK1 knockdown by Western blot analysis. **(C)** mRNA levels of *ELK1, NT5E, NEDD9*, and *PXN* after knockdown of ELK1 (siELK1) or control siRNA (siCtrl) in PC- 3M-luc2 analyzed by qRT-PCR. **(D)** Migration assay of PC-3M-luc2 overexpressing PRK1 or control treated with either siRNA against ElK1 (siELK1) or unrelated control siRNA (siCtrl). **(E)** PRK1 overexpression and ELK1 knockdown was verified by Western blot analysis with the indicated antibodies. β-Actin was used as loading control. (A, D) Cell indices and relative velocities are shown. *n* ≥ 3. Error bars represent ± SD or + SD. ** *p* ≤ 0.01, *** *p* ≤ 0.001.

The ELK1 transcription factor is known to be an effector of active p38-signaling [15, 27]. Since our data showed that p38-signaling is downstream of PRK1 we were interested to analyse the migratory behavior of PC-3M-luc2 cells upon ELK1 depletion. Cells treated with siRNA against ELK1 revealed dramatically reduced migration versus control transfected cells (Figure 5A and 5B). Concordantly, expression of the PRK1 signature genes *NT5E*, *NEDD9*, and *PXN* was downregulated upon ELK1 depletion (Figure 5C). Next, we performed migration assays with PC-3M-luc2 overexpressing PRK1 in presence or absence of ELK1. Upon depletion of ELK1, PRK1 overexpression was no longer able to increase migration (Figure 5D and 5E), supporting that PRK1 signals via ELK1 to control migration. Taken together, our data demonstrate that PRK1 signaling and migratory control is, at least in part, mediated at the transcriptional level by the p38-effector ELK1.

### PRK1 and SPAG9 are overexpressed in human prostate cancer tissue

**Figure 6:**
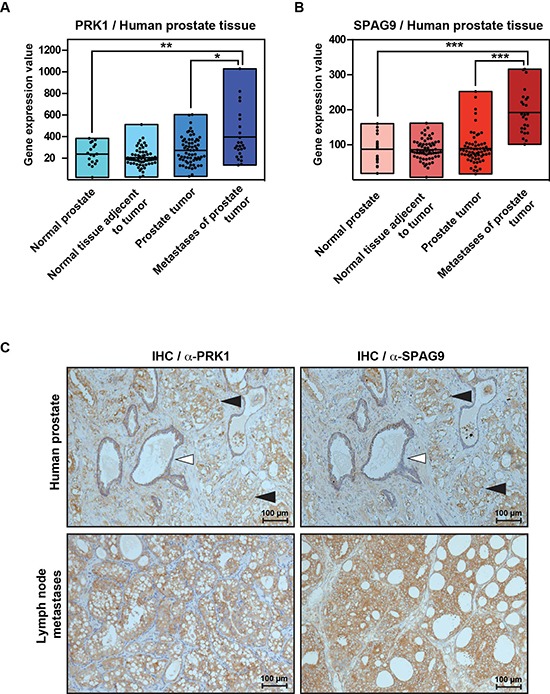
PRK1 and SPAG9 are overexpressed in human prostate cancer tissue **(A)** Analysis of gene expression level of PRK1 and SPAG9 in different human tissue samples (normal prostate *n* = 18, normal tissue adjacent to tumor *n* = 63, prostate tumor *n* = 65, metastases *n* = 25. Raw data provided by Chandran et al. [29] and Yu et al. [28]. **(B)** Overlap of PRK1 and SPAG9 in immunhistochemistry shown in benign prostate tissue (white triangle), prostate cancer (predominantly Gleason 4 pattern) (black triangle) or affected lymph node metastases.

To uncover a potential biological relevance, we analyzed the expression level of PRK1 and SPAG9 in different human tissues. Our analysis of the reported expression pattern of 152 humane prostate tissue samples [28, 29] revealed that both, PRK1 and SPAG9 were significantly overexpressed in prostate cancer tissue, but most importantly also in metastases compared to normal tissue (Figure 6A and 6B). Immunohistochemistry performed on prostate cancer tissue and lymph node metastases sections showed similar distribution of PRK1 and SPAG9 (Figure 6C), indicating their relevance in prostate cancer progression.

### PRK1 depletion or treatment with PRK1-inhibitor Lestaurtinib reduced metastases in an orthotopic prostate mouse model

**Figure 7:**
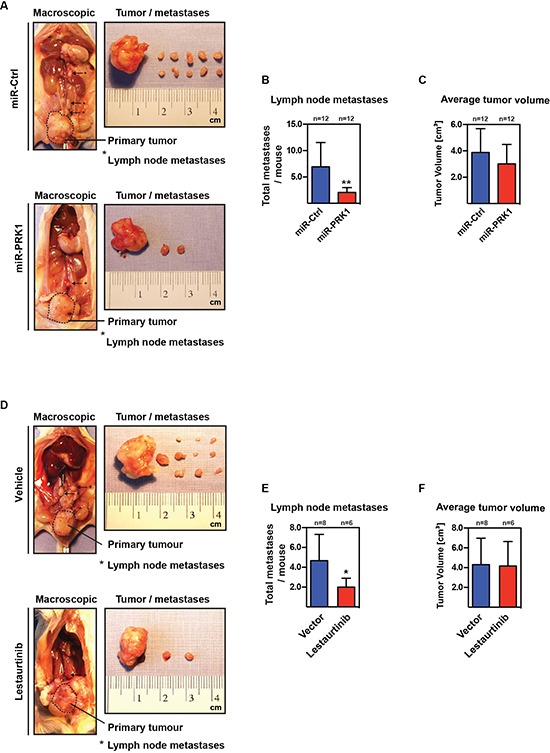
PRK1 controls metastases in an orthotopic prostate mouse model **(A)** Macroscopic view of primary tumour and lymph node metastases of immunodeficient mice injected either with PRK1 depleted PC-3M-luc2 cells (miR-PRK1) or control transfect cells (miR-Ctrl) into the dorsal prostate lobe. **(B, C)** Number of developed lymph node metastasis (B) and primary prostate tumor volume (C) is shown 28 days after injection. **(D)** Macroscopic view (mice, primary tumors, lymph node metastasis) from orthotopic prostate metastases assay with wildtype PC-3M-luc2 cells and subsequent daily treatment of mice with PRK1 inhibitor Lestaurtinib or vehicle. **(E, F)** Number of developed lymph node metastasis (E) and primary prostate tumor volume (F) is shown from both groups 28 days after injection. Statistics were done using Fisher′s exact test, error bars represent + SD. ** *p* ≤ 0.01, * *p* ≤ 0.05.

Our combined *in vivo* and *in vitro* data suggest that metastasis of androgen-independent PCa strongly depends on PRK1. Because in patients metastases arise from a primary tumor we used a second *in vivo* metastasis model. In this orthotopic prostate tumor model, PC- 3M-luc2 cells, either stably expressing miRNA targeting PRK1 or control miRNA, were injected into the dorsal lobe of the prostate of immunodeficient mice. Five weeks after injection, primary tumor size and number of pelvic and retroperitoneal lymph node metastases were analyzed. Importantly, decreased levels of PRK1 led to a significantly decreased number of lymph node metastases compared to the control group (Figure 7A and 7B, Supplemental Figure 5A and 5B). In comparison, tumor volumes were not significantly different (Figure 7C). Tumors as well as lymph node metastases were verified by histological staining (Supplemental Figure 5C). Next, we asked whether enzymatic inhibition of PRK1 also influences development of metastases. Prostate primary tumors were generated by injection of wildtype PC-3M-luc2 into the dorsal lobe of the prostate in immunodeficient mice. After 5 weeks with daily treatment of mice with the PRK1 inhibitor Lestaurtinib we observed a significant reduction of number of metastases compared to vehicle-treated mice (Figure 7D and 7E, Supplemental Figure 5D and 5E). Again, primary tumor volumes in both groups were not altered (Figure 7F). Histological staining confirmed primary tumors and lymph node metastases (Supplemental Figure 5F). Thus, in summary, our data highlight the potential of PRK1 inhibition to prevent androgen-independent prostate cancer metastasis.

## DISCUSSION

In the present study, we uncover a novel mechanism for PRK1-controlled migration of androgen-independent PCa. We show that PRK1 controls migration and invasion but not proliferation of PC-3M-luc2 cells. Genome-wide analysis reveals 1174 PRK1-regulated genes including *PXN*, *NT5E*, and *NEDD9*. These PRK1-regulated genes are required for migration and were therefore chosen as “signature” to read out further analysis of PRK1 signaling. Using these “signature genes” we demonstrate a signaling cascade initiated by the cytokine TGFβ1, via PRK1 and its scaffold protein SPAG9 to the activation of the MAP kinase p38 and eventually to the transcriptional regulator ELK1. We show that both PRK1 and SPAG9 are required for p38 MAP kinase activation and that the nuclear p38 effector ELK1 mediates the migratory effect of PRK1 on gene expression. In mice, the PRK1 inhibitor Lestaurtinib as well as PRK1 depletion leads to a dramatic reduction of metastases underlining the importance of PRK1 for metastasis of androgen-independent prostate cancer cells.

Our analysis uncovers for the first time the transcriptome controlled by PRK1 in androgen-independent PCa cells. Among the PRK1-regulated genes we prototypically describe the targets NT5E, NEDD9, and PXN. NT5E mediates escape of tumor cells from the natural immune system in various cancer types [12, 30]. The integrin adaptor NEDD9 impairs migration and invasion in breast cancer and cervical cancer [31, 32]. Overexpression of PXN increases cell migration and/or metastasis in various cancer types including PCa [33].

As recently shown, migration in PC-3M or PC3 cells can be impaired by manipulation the AMPK-pathway (adenosine monophosphate-activated protein kinase), by overexpression of miR-125a3p, or overexpression of Lysine-specific demethylase 1 (LSD1) resulting in impaired migration [34–36].

Regulation of actin reorganization, stimulation of actin stress fiber depolymerization, and membrane ruffling is orchestrated, in part, by Rho-family-effectors which include PRK1-3 [5, 37]. The isoforms of PRK differ in their enzymatic properties and tissue distribution [37]. Interestingly, after knockdown of PRK3, metastasis in an orthotopic PCa mouse model was blocked and the PPK3-RhoC-complex was highlighted as therapeutic target in late-stage malignancies. However, target genes of the PKN3-RhoC-complex were not described so far [38].

We have identified SPAG9 as a new interacting partner of PRK1, both are required for activation of p38 signaling. We furthermore delineate that TGFβ1 acts via PRK1/SPAG9 to activate p38 thereby controlling expression of migratory-relevant genes. SPAG9 functions as a scaffold protein, which can activate p38 signaling but so far has not been linked with PRK1 [16, 39]. Expression of SPAG9 in epithelial ovarian, breast, cervix, and thyroid cancer, as well as in renal cell and colorectal carcinoma was linked to tumor progression and migration [40], whereas the role of SPAG9 in prostate cancer remained unknown.

p38 MAPK is activated by environmental stress and inflammatory cytokines, such as TGFβ1 [41]. p38 regulates different aspects of cell migration and tumor initiation [15, 42, 43]. Our data show that in PC-3M-luc2 cells TGFβ1-induced migration requires signaling via PRK1 and SPAG9 to activate p38, supporting the idea that PRK1 act as upstream kinase in the p38 MAPK pathway [37]. The data are consistent with reports indicating that PRK1 signals to p38 following alpha1-adrengic receptor activation during smooth muscle differentiation [23, 25]. Amongst others, ELK1 is one of the key effectors of p38 signaling and regulates genes involved in migration, invasion, and metastasis in prostate cancer [20, 21]. ELK1 depletion hinders migration of androgen-independent PCa cells. Interestingly, ELK1 is obligatory for androgen receptor-dependent growth and survival of prostate cancer cells, [44]. Our data now extend the influence of ELK1 on androgen-independent PCa migration.

Our studies might have important implications or future strategies of hormone-resistant PCa. By using androgen-independent PC-3M-luc2 cells we were able to highlight a novel therapeutic strategy by blocking PRK1, completely independent of AR-controled network pathways. Blocking PRK1 could be beneficial, in case of life threatening metastases from advanced primary tumor and for men with already metastatic PCa to diminish disseminated visceral metastases. Our *in vivo* data demonstrate the efficiency of the kinase inhibitor Lestaurtinib in mice to reduce metastases arising from primary tumor. Lestaurtinib, structurally related to staurosporine, was reported to inhibit TrkA, TrKb, TrKC, JAK, and FLT3 [45]. Due to the strong activity against FLT3 Lestaurtinib was first clinically studied in acute leukemia, with a considerable response rate of 27% [46]. Recently, Lestaurtinib has been established as a potent PRK1-inhibitor *in vitro* and *in vivo* [22]. A phase II study (NCT00081601) of CEP-701 (Lestaurtinib) was designed to assess the activity of CEP-701 in patients with hormone refractory recurrent PCa. The study include a termination rule if no response (i.e., PSA decline >50% from baseline) was observed in any of nine patients after 12 weeks of therapy [47], which was the case. Interestingly, five of nine patients (55%) had decreases in serum PSA following cessation of CEP-701. The duration of this PSA decrease ranged from two weeks to six months by an average of 38 ± 9% (range 15–60%) from the peak PSA level achieved on the last day of CEP-701 therapy. One patient′s PSA decreased sequentially over a 6 months period almost back to his baseline PSA level measured at the start of CEP-701 therapy [47]. It was concluded that PSA response is an inadequate indicator of response in Lestaurtinib treated PCa patients [47]. Thus, the effectiveness of Lestaurtinib as treatment for prostate cancer has not been adequately tested. Currently, to best of our knowledge there is no ongoing clinical trial for Lestaurtinib in the field of prostate cancer (http://www.cancer.gov/clinicaltrials).

In addition, the screening for novel PRK1-inhibitors may help to find clinically useful substances in the near future [48]. Taken together, our data establish a strong preclinical rational that justifies clinical investigation of PRK1 inhibition in androgen-independent PCa.

## MATERIAL AND METHODS

### Plasmids

Expression plasmids for overexpression PRK1 or SPAG9 were generated by LRII recombination according to the supplier (Gateway, Invitrogen) using entry clones (PRK1: GeneCopoeia: GC-Z5489-CF; accession number NM_213560), (SPAG9: GeneCopoeia: GC-E2655-CF; accession number NM_001130528) and a puromycin-selectable and doxycycline-inducible pRTS plasmid modified according to Zhu et al. [49]. Vectors without insert were used as control.

### Chemical compounds

Lestaurtinib (LC Labortories; Cat. No. L-6307), SB 203580 (CALBIOCHEM; Cat. No. 559395), Ro318220 (AdipoGen; AG-CR1-0111), Tofacitinib/CP-690550 (Santa Cruz; sc-207457), TGFβ-1 (Invitrogen; Cat. No. PHG9204).

### Cell culture and transfection

PC-3M-luc2 cells and Du145 cells were cultured in EMEM (Lonza, 12–125) supplemented with 10% FCS, 1% L-glutamine (Lonza, BE17-605E) and 1 % penicillin-streptomycin (Lonza, DE17-602E). MDA-MB-231 and PANC-1 cells were cultured in DMEN (Lonza, BE12-614F), T778 cells in RPMI 1640 (Gibo, 31870025), each supplemented with 10% FCS, 1% L-glutamine (Lonza, BE17-605E) and 1 % penicillin-streptomycin (Lonza, DE17-602E). RNAi knockdown was performed using Dharmafect 2 (Thermo Scientific, T-2002-01) in PC- 3M-luc2 cells, PANC1 cells and MDA-MB-231 cells, and Dharmafect 4 (Thermo Scientific, T-2004-01) in Du145 cells using 25 nM siRNA (final concentration) according to the manufacturer′s instruction. The following siRNAs were used for RNAi-mediated knockdown:

siCtrl 5′-GAAAGUCCUAGAUCCACACGCAAAU-3′.
siPRK1 5′-GAACAUGAUCCAGACCUACAGCAAU-3′.
sip38 5′-CAGACCATTTCAGTCCATCATTCAT-3′.
siPXN 5′-CATACCCAACTGGAAACCACACATA-3′.
siELK1 5′-CACATCCCTTCTATCAGCGTGGATG-3′.

The following miRNAs were used for stable miRNA expression:

miR-Ctrl 5′-GAAATCGCTGATTTGTGTAGTCGTTTTGG CCACTGACTGACGACT ACACATCAGCGA TTT-3′.
miR-SPAG9 5′-GATTTCTGGTGGTTTATCCATCGTTTTGG CCACTGACTGACGA TGGATACCACCAG AAAT-3′.
miR-NT5E 5′-GTACACGGTGAACCAGATAGTGGTTTTG GCCACTGACTGACCACTATCTTTCACCG TGTA-3′.
miR-NEDD9 5′-GTAATGAGCACAGCCACCATCCGTTTTG GCCACTGACTGACGGATGGTGTGTGCTC ATTA-3′.

### Generating stable miRNA-expressing PC-3M-luc2 cell lines line for metastases assay

Using the BLOCK-iT™ Pol II miR RNAi Expression Vector Kit (Life Technologies; K4935-00), oligonucleotides were ligated into pcDNA6.2™-GW/EmGFP-miR-vector to target gene of interest. The Gateway® BP-Clonase® II Enzyme mix (Life technologies, 11789-020) was used to transfer sequences into the shuttling vector pDONR™ 221-vector (Life technologies, 12536-017). For generation of expression plasmids, the pRTS-plasmid55, a puromycin-selectable and doxycycline-inducible expression vector was modified to contain a Gateway cassette, V5 and His-tag epitope. miRNAs were cloned into this vector using the Gateway LR-clonase® II enzyme mix (Life technologies, 11791-100) according to manufactures instruction. PC-3M-luc2 or DU145 cells were transfected with expression vector or empty control vector using Dharmafect Duo (Thermo Scientific, T-2010-01). Puromycin (Sigma, St Louis, MO, USA, P8733, 5 μg/ml) was administered to cells 24 h post transfection for selection of successfully transfected cells. After reaching the appropriate cell density doxycycline (2 μg/ml) was added 24 h before injection or transplantation of cells into mice.

### Immunofluorescence

Immunofluorescence analysis was essentially performed as described by Müller *et al*. [50]. For primary antibody α-JIP4/SPAG9 (Santa Cruz, sc-271492) and α-PKN1/PRK1 (Epitomics, 2662-1) were used. Specific staining was monitored by using unrelated rabbit-IgG (sc-2027, Santa Cruz) and unrelated mouse-IgG (sc-2025, Santa Cruz). Antibodies conjugated to Alexa-488 (Invitrogen, A-11034, 1:600), Alexa-547 (Invitrogen, A-11030, 1:600) were used as secondary antibodies. Staining was documented with a confocal microscope (Leica SP2AOBS).

**Immunhistochemistry** was performed as described by Metzger et al. [50]. α-JIP4/SPAG9 (Santa Cruz, sc-271492) and α-PKN1/PRK1 (Epitomics, 2662-1) were used 1:300 and 1:600.

### Western blot analysis and immunoprecipitation

Experiments were performed as described12. The following antibodies were used for western blots: α-β-Actin (Sigma, A1978), α-α-Tubulin (Sigma, T6074), α-PKN1/PRK1 (Epitomics, 2662-1), α-PXN (Cell Signaling, 2542), α-phospho38 MAPK (Thr180/Tyr182) (Cell Signaling, 4631), α-p38 (Santa Cruz sc-535), α-ELK1 (abcam ab32106), α-JIP4/SPAG9 (Santa Cruz, sc-271492).

### Apoptosis assay

The apoptotic fraction of the cells was determined by measuring active caspase 3 with the Caspase3/CPP32 Colorimetric Protease Assay Kit (Invitrogen). Briefly, the cells were harvested, lysed, and centrifuged. 50 μg of the supernatant were used for the apoptosis assay. Resulting absorbance was measured at 405 nm. T778 liposarcoma cells treated with 10 μM Nutlin-3a (Cayman) served as a positive control [51]. siRNA-treatment in PC-3M-luc2 cells or Nutlin-3a treatment in T778 cells was applied 24 h before starting the assay.

### Sucrose density centrifugation

Sucrose was dissolved in 50 mM Tris, 100 mM NaCl, and Complete Protease Inhibitor (EDTA-free, Roche). Whole cell lysate was added to the 40 – 10 % sucrose gradient and centrifuged at 185.000 g for 16 hours. 17 fractions were collected and immunoblotted with the indicated antibodies.

### Cell proliferation, migration, and invasion assays

Prior to the experiment PC-3M-luc2 cells were treated for 24 hours with siRNA or for 48 h with doxycyclin (2 μg/ml), respectively. Proliferation, cell migration, and invasion were monitored using the xCelligence system (Roche). For invasion, transwell chamber filters (Roche) were coated with matrigel (BD Biosciences, 354230) diluted 1:40 in EMEM medium. For proliferation 5 × 10^3^ PC-3M-luc2 cells, 1 × 10^4^ and Du145 cells, were seeded into E-plate 16 (Roche). For migration and invasion PC-3M-luc2 cells were seeded at 5 × 10^4^, Du145 cells at 1 × 10^4^ in to the transwell containing 0.5 % FCS EMEM in the upper chamber and 10 % FCS EMEM in the lower chamber. For migration assay of MDA-MB-231 and PANC-1 cells were seeded at 8 × 10^4^ in to the transwell containing 0.5 % FCS DEMEM in the upper chamber and 10 % FCS DMEM in the lower chamber. Cell indices were automatically recorded every 15 minutes. Relative velocities represent the change of the cell index over time. Chemicals compounds were added the time point of starting the migration assay at the following concentrations: Lestaurtinib (25 μM), SB 203580 (20 μM), Ro318220 (20 μM), Tofacitinib/CP-690550 (1nM-10 μM). TGFß1 was added immediately before starting the assay to over night serum starved cells at a concentration of 2 ng/ml.

### RNA extraction and semiquantitative RT-PCR

RNA isolation and quantitative PCR after reverse transcription were performed as described [36]. For normalisation of expression in PC-3M-luc2 and Du145 cells *ACTB*, *HPRT1*, and *POLR2A* were used and data were related to negative control cells treated with either control siRNA, empty Vector or empty virus. Experiments were repeated in triplicate at least three times. Primers are shown in Supplementary Table 1.

### RNA sequencing (RNA-seq)

RNA samples were sequenced by the standard Illumina protocol to create raw sequence files (fastq files). The reads were aligned to the hg19 build of the human genome using TopHat [52]. Gene expression values were generated for RefSeq annotated transcripts using HOMER software [53] and differential expression calculation was performed using EdgeR [54] and DESeq version [55].

### *In vivo* metastasis Assay

All mice were housed in the pathogen-free barrier facility of the University Medical Center Freiburg in accordance with institutional guidelines and approved by the regional board. 7–8 weeks old male C.B-17.Cg-Prkdc^scid^ Lyst^bg^/Crl-mice (Charles River) were used for *in vivo* metastasis assays. 1 × 10^6^ PC-3M-luc2 cells were resuspended in 100 μl PBS or 20 μl Matrigel and injected either into the lateral tail vein or the dorsal lobe of the mouse prostate. For bioluminescent imaging mice were anesthetized with isofluorane (Forene®, Abbott GmbH) and 150 μg/g D-Luciferin (Caliper Life Science, 119222) were applicated intraperitoneal. Two minutes after injection bioluminescence was imaged for 30 sec to 5 min at day 7, 14, 21, and 28 of the experiment. At day 28 mice were euthanized and organs with luminescent signals were collected for H&E staining. For the orthotopic metastasis model primary tumors and axillary lymphnodes were dissected after 28 days. Volume [mm^3^] of primary tumors was calculated as follows: ¾ × π × diameter 1 [mm] × diameter 2 [mm] × diameter 3 [mm].

### Statistical analysis

If not otherwise stated, significance was calculated using an unpaired t-test. Data are calculated as mean ±SEM or +SEM.

## SUPPLEMENTARY FIGURES AND TABLES


