# Supplementary material for: PRK1/PKN1 controls migration and metastasis of androgen-independent prostate cancer cells
Source: Oncotarget. 2014 Dec 10;5(24):12646–64. doi: 10.18632/oncotarget.2653 (PMC4350344; doi:10.18632/oncotarget.2653)
Supplement: Supplementary file 1 [file oncotarget-05-12646-s001.pdf]

SUPPLEMENTARY FIGURES AND TABLES

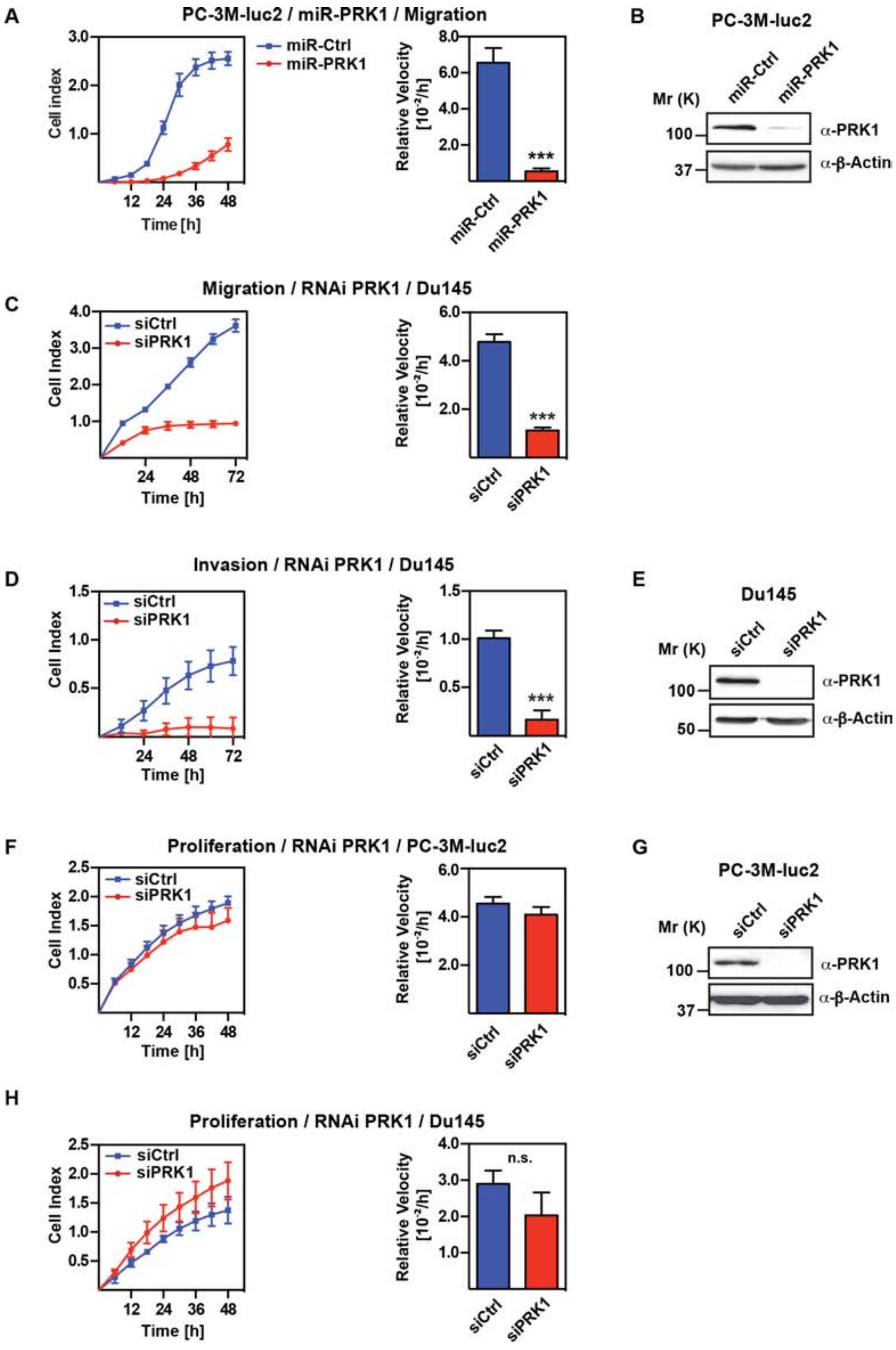

Supplementary Figure S1: Behavior of cancer cells upon loss or gain of PRK1. **(A)** Migration assay of PC-3M-luc2 cells stably transfected with miRNA targeting PRK1 (miR-PRK1) versus control (miR-Ctrl). Migration **(C)** and invasion **(D)** assays of Du145 cells treated with siRNA against PRK1 (siPRK1) or unrelated siRNA (siCtrl). **(F, H)** Proliferation assay of PC-3M-luc2 and Du145 cells upon treatment with siRNA against PKR1 (siPRK1) or unrelated siRNA (siCtrl).

(*Continued*)

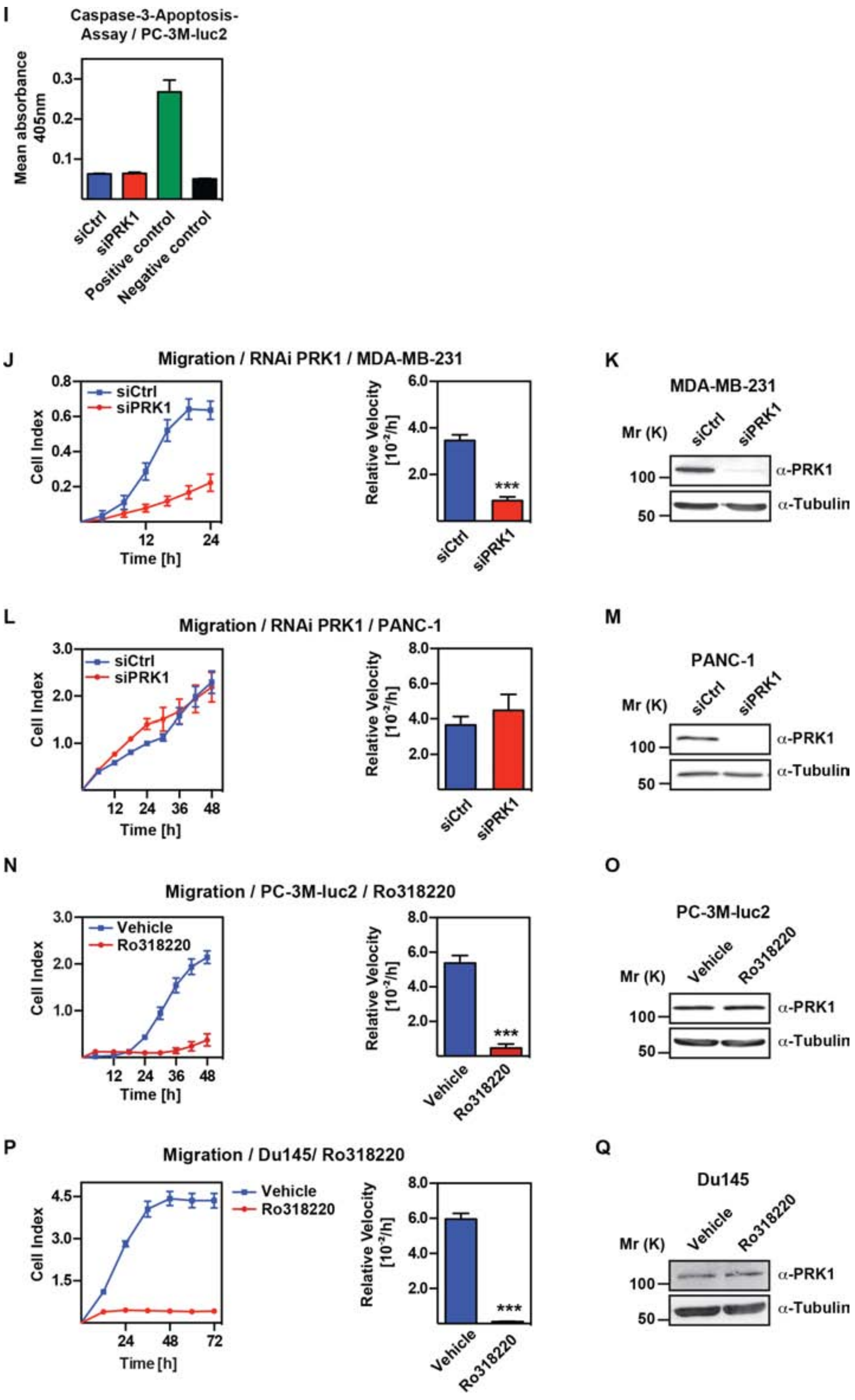

Supplementary Figure S1 (*Continued*): (I) Analysis of apoptosis upon PRK1 knockdown. Caspase-3 apoptosis assay of PC-3M-luc2 cells after treatment with siRNA against PRK1 (siPRK1) or control siRNA (siCtrl). T778 cells treated with 10µM Nutlin-3a (24 h) served as a positive control. siRNA-treatment or Nutlin-3a treatment was applied 24 h before starting the assay. **(J)** Migration assay of MDA-MB-231 cells treated with siRNA against PRK1 (siPRK1) or unrelated siRNA (siCtrl). **(L)** Migration assay of PANC-1 cells treated with siRNA against PRK1 (siPRK1) or unrelated siRNA (siCtrl). **(N, P)** Migration assay of (N) PC-3M-luc2 or (P) Du145 cells cells treated with 30 µM PRK1 inhibitor Ro318220 or vehicle.

(*Continued*)

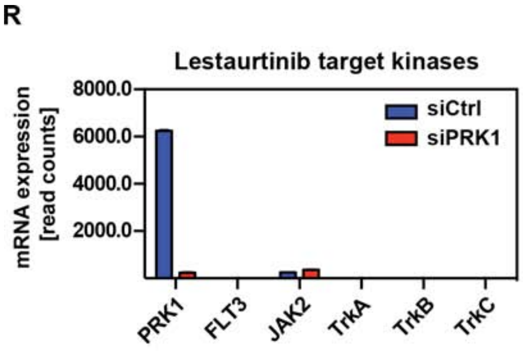

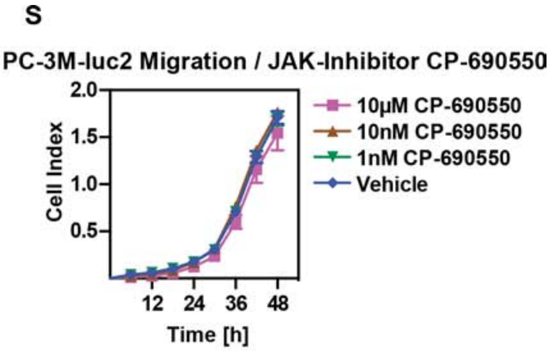

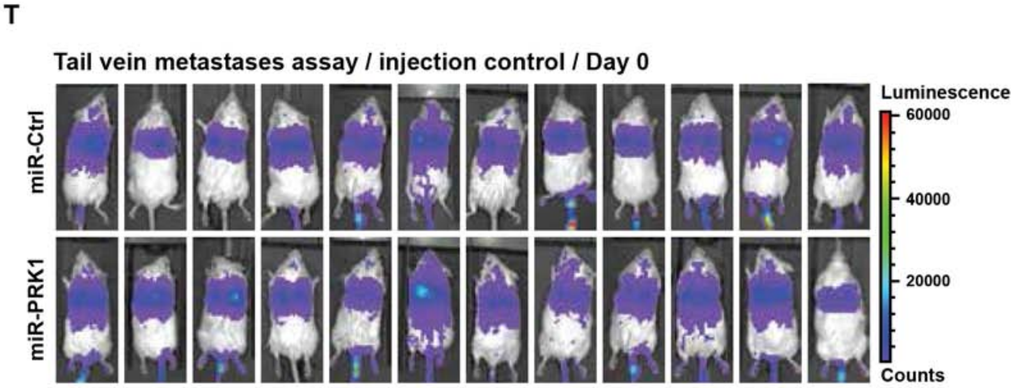

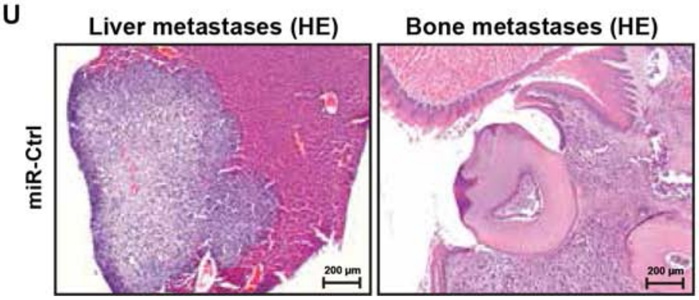

Supplementary Figure S1 (*Continued*): (R) Mean read counts from RNA-Seq. showing mRNA-expression of PRK1, FLT3, JAK2, TrkA, TrkB, and TrkC in PC-3M-luc2 cells treated with either siRNA against PRK1 (siPRK1) or unrelated siRNA (siCtrl). **(S)** Migration assay of PC-3M-luc2 cells treated with the JAK-Inhibitor CP-690550 (1 nM-10 µM) or vehicle. **(T)** PRK1 controls metastases in a mouse model. Injection of equal amounts of cells into the tail vein of immunodeficient mice was confirmed by bioluminescence. **(U)** *Ex vivo* confirmation of metastases 28 days after tail vein injection of PC-3M-luc2 cells by H&E staining. (A, C, D, F, H, J, L, N, P, S) Cell indices and relative velocities are shown. $n \geq 3$. Error bars represent ± SD or + SD. *** $p \leq 0.001$. **(B, E, G, K, M, O, Q)** Verification of PRK1 levels in different cell lines by Western blot analysis decorated with the indicated antibodies. β-Actin was used as loading control.

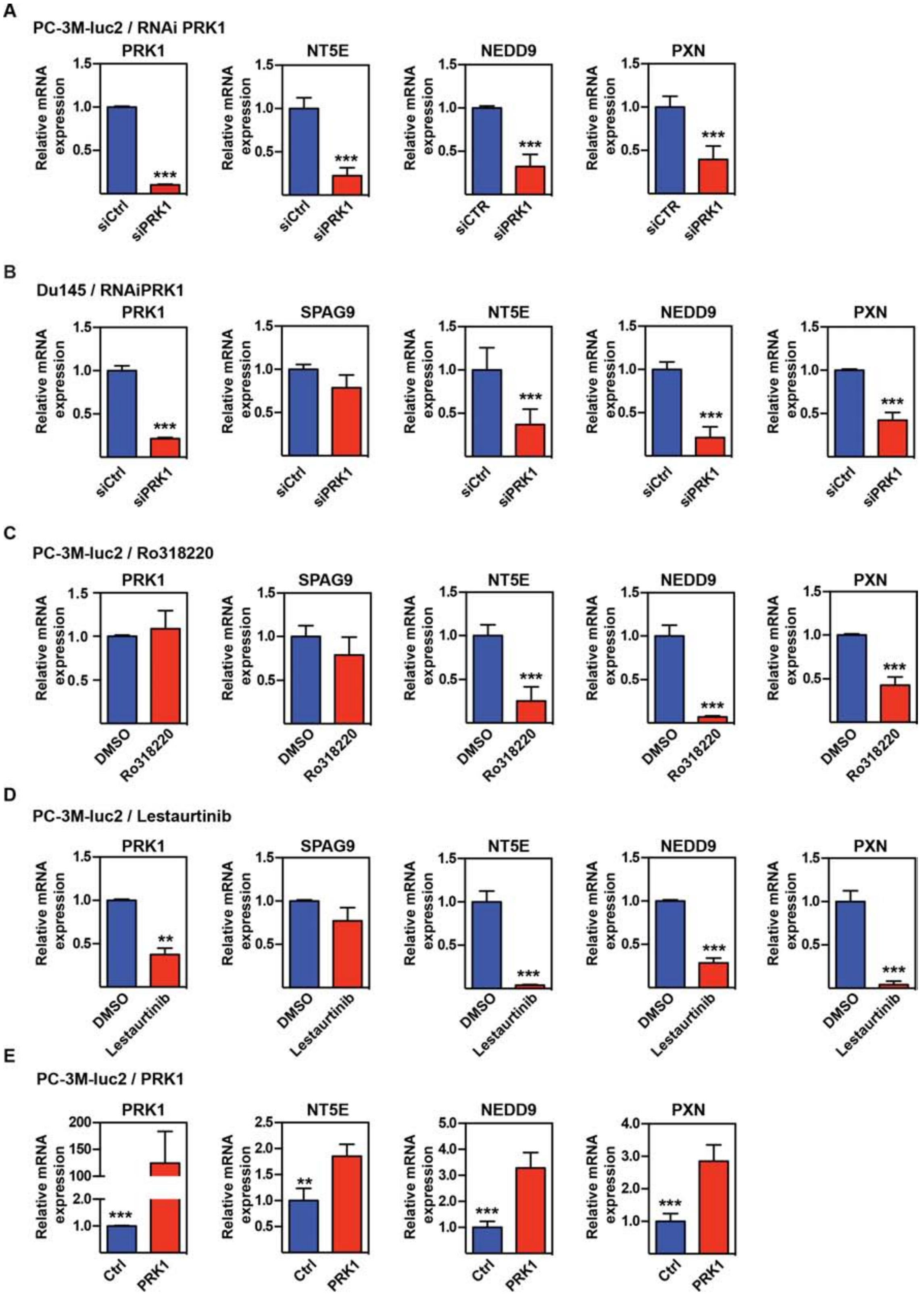

Supplementary Figure S2: NT5E, NEDD9, and PXN are regulated by PRK1 in PC-3M-luc2 and DU145 cells. mRNA levels of *PRK1*, *SPAG9*, *NT5E*, *NEDD9*, and *PXN* were analyzed by qRT-PCR upon either **(A)** PRK1 depletion in PC-3Mluc2 cells or **(B)** Du145 cells, or in PC-3M-luc2 **(C)** upon treatment with 30 µM Ro318220, or **(D)** with 25 µM Lestaurtinib, or **(E)** upon PRK1 overexpression versus controls.

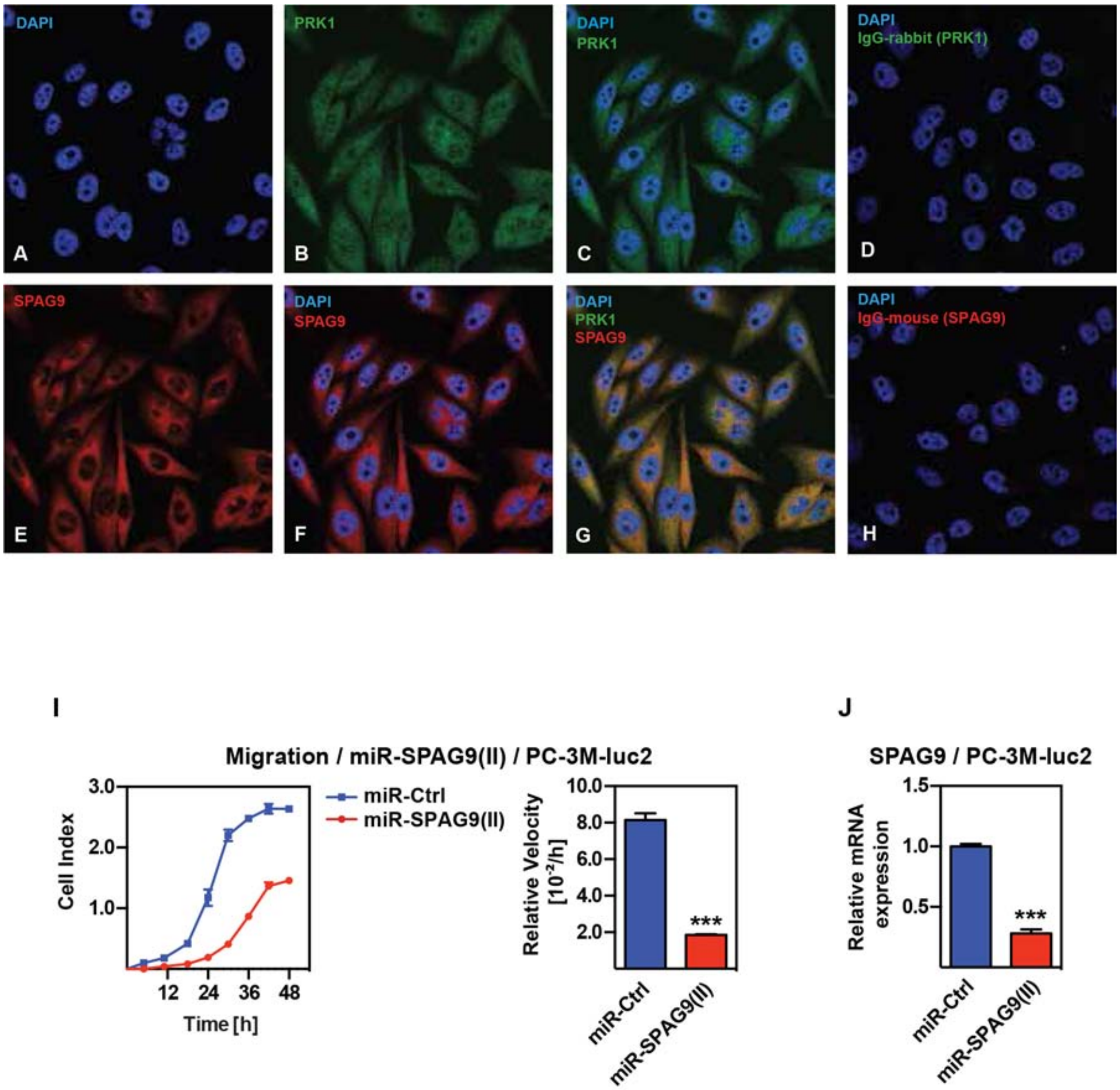

Supplementary Figure S3: Colocalization of PRK1 and SPAG9 in the cytoplasm of PC-3M-luc2 cells. **(A)** DAPI-staining of DNA (blue), **(B)** cytoplasmatic PRK1 staining (green) and **(C)** overlay. **(E)** SPAG9 staining (red), and **(F)** overlay with DAPI. **(G)** Overlay of PRK1 (green) and SPAG9 (red) with DAPI (blue). **(D)** IgG-rabbit for PRK1 or **(H)** IgG-mouse for SPAG9 was used as controls. **(I)** Migration assays of PC-3M-luc2 cells stably expressing a second miRNA against SPAG9 (miR-SPAG9 (II)) or control (miR-Ctrl). Cell indices and relative velocities are shown. $n \geq 3$. Error bars represent ± SD or + SD. **(J)** SPAG9 knockdown was verified by qRT-PCR.

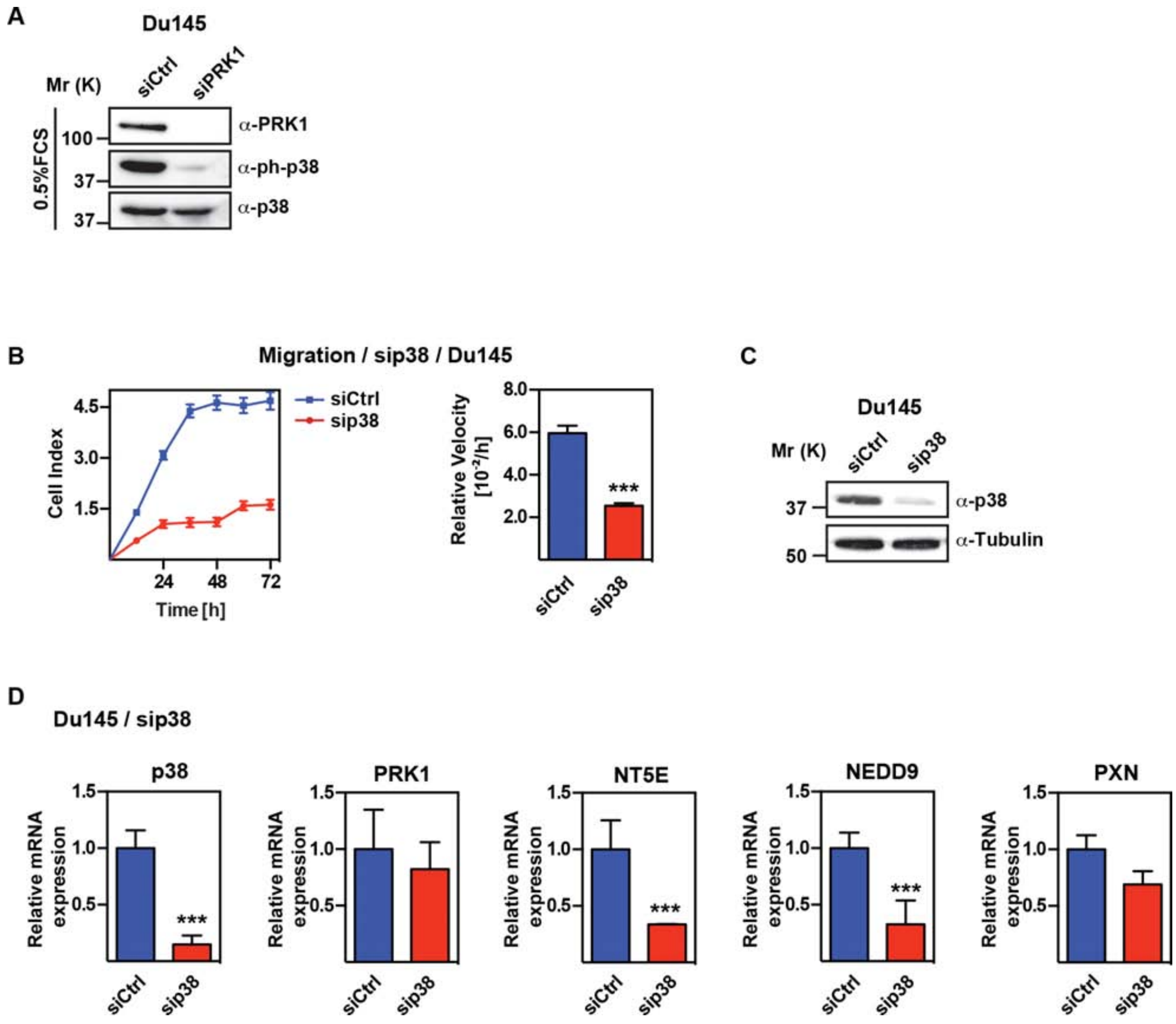

Supplementary Figure S4: PRK1 determine phosphorylation status of p38 in Du145. **(A)** Western blots showing levels of phospho-p38 (ph-p38) upon knockdown of PRK1. Level of phospho-p38 was analyzed in Western blots with the indicated antibodies .Total amount of p38 protein is shown as control. **(B)** Migration assay of Du145 cells treated either with siRNA against p38 (sip38) or unrelated control siRNA (siCtrl). Cell indices and relative velocities are shown. $n \geq 3$. **(C)** Efficiency control of p38 knockdown was performed by Western blot analysis. **(D)** mRNA levels of *PRK1*, *NT5E*, *NEDD9*, *PXN*, and *p38* upon knockdown of p38 in Du145 cells were analyzed by qRT-PCR. Error bars represent ± SD or + SD. *** $p \leq 0.001$.

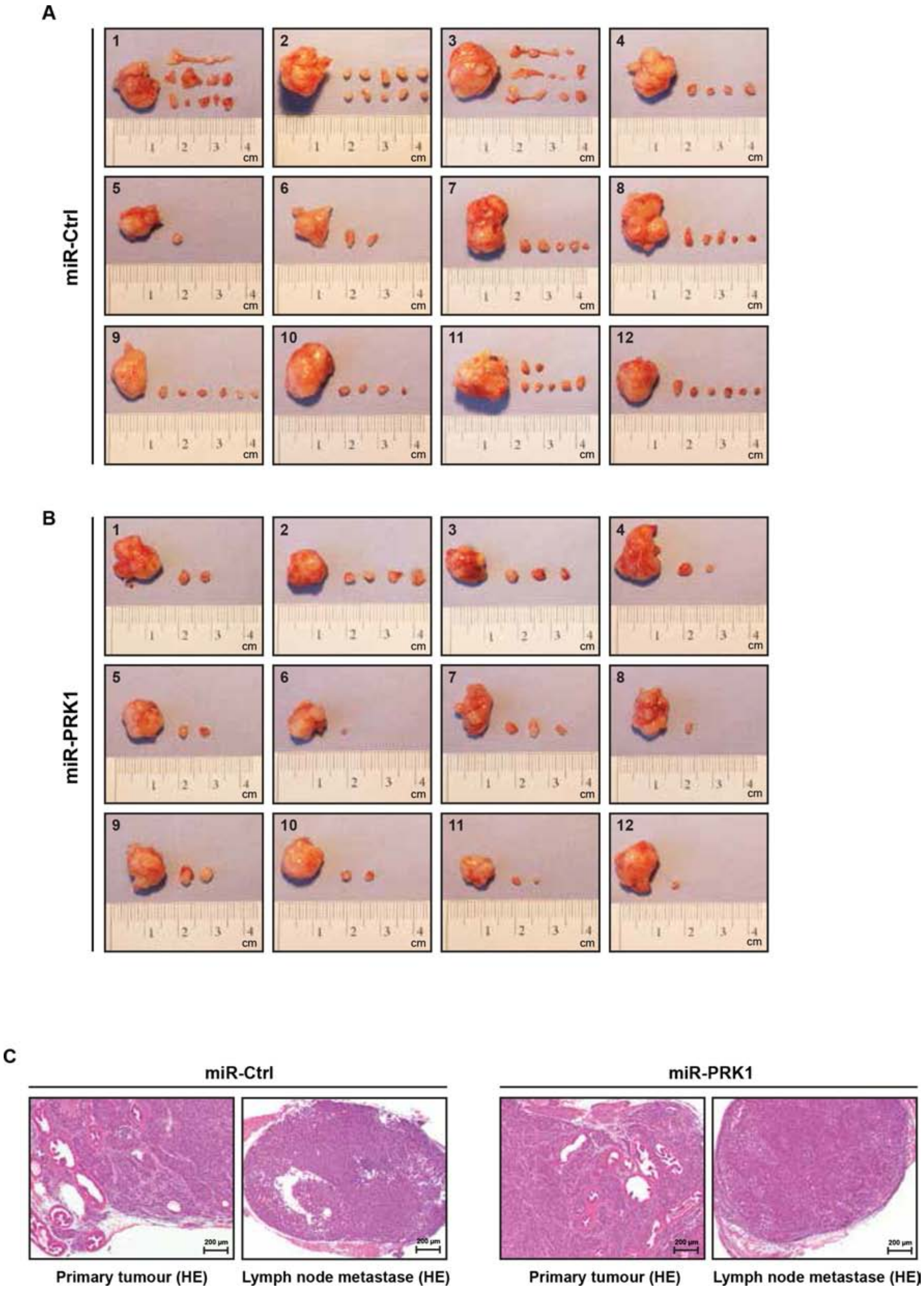

Supplementary Figure S5: PRK1 controls metastasis *in vivo*. **(A, B)** Primary tumors and lymph node metastases in an orthotopic tumor model 5 weeks after injecting PC-3M-luc2 transfected with (A) control miRNA (miRNA) or (B) miRNA targeting PRK1 (miRNA-PRK1) in the dorsal lobe of the mouse prostate.

(*Continued*)

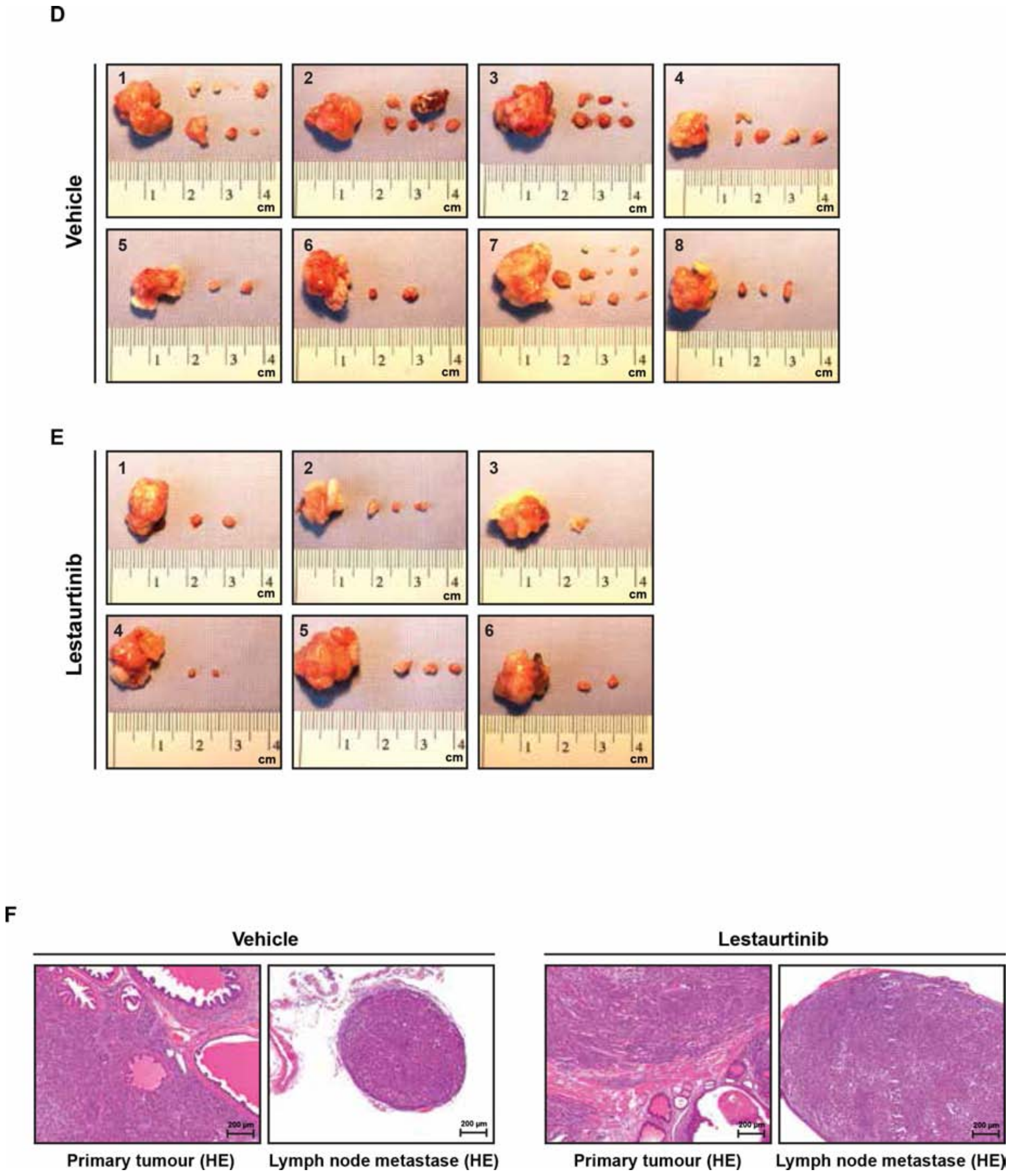

Supplementary Figure S5 (*Continued*): (D, E) Primary tumors and lymph node metastases 5 weeks after injecting PC-3M-luc2 cells into the dorsal lobe of the mouse prostate followed by daily treatment with (D) vehicle or (E) with Lestaurtinib (15 mg/kg). **(C, F)** Representative H&E-stainings of the primary tumors and corresponding lymph node metastases from orthotopic metastases model.

Supplementary Table S1. Primers used for qRT-PCR

	Fwd	Rev
PRK1	CAGGGGATGATGAGGAGGAG	gaagaagggctgtttcttcaca
PXN	ATCCTGAGTGCTTTGTGTGC	CAGAAGGCACAGACGAAGTG
SPAG9	CAAATGGTGCTTCTCCAGTG	TGGGATCTGAACTCCCAAAG
NT5E	GAAGTTGTGGGAATCGTTGG	TCCATTTCAAAACCCGAATG
NEDD9	AGGAGGAGTTTGAGAGGCAAC	ACTGTTTGTGGTGGGTAGGC
P38/MAPK14	GGCACACAGATGATGAAATGAC	CAATGTTCTTCCAGTCAACAGC
ELK1	CTTCTGGAGCACCCTGAGTC	agagcatggatggagtgacc
ACTB	GTGGCATCCACGAAACTACC	GTACTTGCGCTCAGGAGGAG
POL2A	GCACCACGTCCAATGACAT	GTGCGGCTGCTTCCATAA
HPRT	CCTGGCGTCGTGATTAGTGAT	AGACGTTCAGTCCTGTCCATAA
